# Antimicrobial activity and mechanistic insights into the tick peptide persulcatusin against vancomycin-susceptible and vancomycin-resistant *Enterococcus faecium* strains

**DOI:** 10.3389/fmicb.2026.1834116

**Published:** 2026-05-04

**Authors:** Ikki Morozumi, Shuichi Nakamura, Naruhide Miyoshi, Moe Narita, Nobumichi Kobayashi, Ken Kikuchi, Emiko Isogai, Takashi Sasaki

**Affiliations:** 1 Department of Animal Microbiology, Graduate School of Agricultural Science, Tohoku University, Sendai, Japan; 2 Department of Applied Physics, Graduate School of Engineering, Tohoku University, Sendai, Japan; 3 Division of Hygiene, Department of Social Medicine, Sapporo Medical University School of Medicine, Sapporo, Japan; 4 Department of Infectious Diseases, Tokyo Women’s Medical University, Shinjuku, Japan; 5 Animal Research Center, Sapporo Medical University School of Medicine, Sapporo, Japan

**Keywords:** antimicrobial peptide (AMP), *Enterococcus faecium*, persulcatusin (IP), vancomycin, vancomycin-resistant *E. faecium* (VREfm)

## Abstract

**Introduction:**

The emergence of multidrug-resistant bacteria, particularly vancomycin-resistant enterococci (VRE), represents a critical threat to global public health. Therefore, the development of novel therapeutic strategies is urgently required. Antimicrobial peptides (AMPs) are promising candidates for combating these infections. Persulcatusin (IP), an AMP from the hard tick *Ixodes persulcatus*, exhibits potent activity against *Staphylococcus aureus* via membrane disruption. However, IP’s efficacy and mechanism against enterococcal species remain unclear. This study aimed to evaluate the antibacterial potency of IP against clinical isolates of *E. faecalis* and *E. faecium*, including vancomycin-resistant strains, and determine whether its mode of action involves membrane disruption, as observed in S. aureus.

**Methods:**

To assess the antibacterial efficacy of IP against enterococcal strains, including VRE, we determined the minimum inhibitory concentrations (MICs) and conducted short-term killing assays using clinical isolates of *E. faecalis* and *E. faecium*. Furthermore, we conducted electron microscopic observation of bacterial morphology, intracellular localization analysis using 5-carboxyfluorescein-labeled IP, and DNA-binding assays to elucidate its mechanism.

**Results:**

IP exhibited the lowest MIC value (1.25 μg/mL) against vancomycin-susceptible *E. faecium* among the five AMPs tested (range: 10 to >40 μg/mL), showing growth-inhibitory activity comparable to that of vancomycin (1.25 μg/mL). IP also effectively inhibited the growth of vancomycin-resistant *E. faecium* (VREfm) strains with an MIC of 0.625 μg/mL. Conversely, IP showed no growth inhibition against any tested *E. faecalis* strains, revealing species-specific selectivity. Unlike the effects on *S. aureus*, IP did not induce membrane damage in Enterococcus strains. Instead, the growth-inhibitory activity of IP correlated with its cellular penetration capability (in the order: *S. aureus > E. faecium > E. faecalis*). Additionally, IP demonstrated high binding affinity to bacterial DNA.

**Discussion:**

This study is the first to demonstrate that IP effectively inhibits the growth of *E. faecium*, including VREfm, through a mechanism distinct from its known membrane-disrupting action. Given its high cellular penetration and DNA-binding capacity, the antimicrobial action of IP against *E. faecium* is likely to involve an intracellular target, such as genomic DNA. Our results highlight IP as a promising therapeutic agent for treating challenging VREfm infections.

## Introduction

Enterococci are Gram-positive commensal bacteria that are commonly found in the gastrointestinal tracts of humans and other mammals. They also inhabit diverse environmental niches, including soil, surface water, sewage, and plants, which contributes to their remarkable capacity to acquire and disseminate antibiotic resistance. While some species play a beneficial role in food fermentation and preservation (Franz et al., 2011), others have transitioned into serious clinical threats. While both *Enterococcus faecium* and *E. faecalis* are leading causes of nosocomial infections worldwide, the prevalence of *E. faecium* has risen at an alarming rate over the past two decades (Arias and Murray, 2012; Ramsey et al., 2014; Tacconelli et al., 2018a; Liese et al., 2019; Schroder et al., 2020; Wei et al., 2024). These two enterococcal species are responsible for a wide range of clinical manifestations, most notably urinary tract infections, soft tissue infections, bacteremia, device-associated infections, and infective endocarditis (Arias and Murray, 2012; Garcia-Solache and Rice, 2019). Importantly, most clinical *E. faecium* isolates are multidrug-resistant, and these strains are characterized by a high prevalence of resistance to β-lactams, a pattern that is not typically observed in *E. faecalis* (Garcia-Solache and Rice, 2019).

For decades, the glycopeptide antibiotic vancomycin has been used to treat enterococcal infections. However, this clinical reliance is now under threat due to the alarming global spread of vancomycin-resistant enterococci (VRE), particularly vancomycin-resistant *E. faecium* (VREfm) (Panesso et al., 2010; Sacramento et al., 2017). Due to its frequent resistance to multiple antibiotic classes, VREfm has been recently classified by the World Health Organization (WHO) as a priority pathogen for antimicrobial development (Tacconelli et al., 2018a; World Health Organization, 2022, 2024). The resulting scarcity of effective therapeutic options against VREfm underscores a critical and immediate requirement for the development of novel antibacterial agents to address the escalating threat of antimicrobial resistance (AMR).

Antimicrobial peptides (AMPs) are integral components of the innate immune systems of nearly all living organisms and are widely regarded as promising candidates in the ongoing struggle against AMR (Seo et al., 2012; Deshayes et al., 2021; Rima et al., 2021; Ma et al., 2024). The primary mechanism of these peptides involves the disruption of bacterial membranes along with the inhibition of various essential cellular processes, providing them with broad-spectrum activity against a diverse range of pathogens, including bacteria, fungi, viruses, and parasites. Unlike traditional antibiotics, a single AMP can exert bactericidal action through multiple simultaneous pathways. This multi-targeted approach minimizes the opportunity for pathogens to develop several resistance-conferring mutations at once, thereby reducing the likelihood of inducing antimicrobial resistance (Marr et al., 2006; Garvey, 2023; Alzain et al., 2025).

Persulcatusin (IP) is a defensin-like antimicrobial peptide (AMP) identified in the tick *Ixodes persulcatus*, with mRNA transcripts predominantly detected in the midgut (Saito et al., 2009; Isogai et al., 2011). We have previously reported that IP possesses potent antimicrobial activity against Gram-positive bacteria, specifically targeting *Staphylococcus aureus*, including methicillin-resistant *S. aureus* (MRSA) (Miyoshi et al., 2016; Miyoshi et al., 2017). The previous reports revealed that IP can exhibit antibacterial efficacy against *S. aureus* that is superior or comparable to that of vancomycin, suggesting that this activity primarily stems from the disruption of membrane integrity. Notably, while IP induces lower levels of membrane leakage than Nisin, its high potency against staphylococcal strains may result from a secondary mechanism involving additional unknown targets to complement its effects on the membrane. Also, the antimicrobial profile and the mechanism of action of IP against *Enterococcus* species, particularly VREfm, have yet to be explored.

Consequently, the aim of the present study was twofold. First, we evaluated the antimicrobial activity of IP against clinical isolates of *E. faecalis* and *E. faecium*, including VRE strains, using a multifaceted approach. This involved determining minimum inhibitory concentrations (MICs) and time-kill assays for comparisons with other AMPs. Second, we sought to clarify the mechanism of action and the specific cellular targets of IP within these enterococcal species. This involved morphological observation using scanning electron microscopy, intracellular localization analysis with 5-carboxyfluorescein (FAM)-labeled IP, and DNA-binding assays. Our findings suggest that the growth-inhibitory activity of IP extends via DNA-binding to *E. faecium*, including vancomycin-resistant strains, through a process that does not involve membrane disruption.

## Materials and methods

### Bacterial strain and culture conditions

*Enterococcus faecalis* ATCC49533 and *E. faecium* ATCC6057 were used as vancomycin-susceptible *E. faecalis* (VSEfl) and *E. faecium* (VSEfm) reference strains in this study. We also utilized 12 clinical isolates, consisting of vancomycin-resistant *E. faecalis* (VREfl) and VREfm strains (Table 1). The presence of *vanA* or *vanB* genes in these isolates was confirmed by PCR using established protocols (Dutka-Malen et al., 1995; Kariyama et al., 2000; Elsayed et al., 2001). *E. faecalis* VRE.fl-1 (*vanA*-positive) and *E. faecium* VRE.fm-1 (*vanA*-positive) strains were used for further experimentation as representative strains. All enterococcal strains were cultured in brain heart infusion (BHI) broth (Shimadzu Diagnostics Corporation, Tokyo, Japan), with or without 4 μg/mL vancomycin, and incubated for 18 to 19 h at 37 °C. For comparative analysis, the vancomycin-resistant *S. aureus* (VRSA) strain VRS1 was grown in Trypto-Soya (TS) broth (Nissui Pharmaceutical, Tokyo, Japan) supplemented with 4 μg/mL vancomycin for 18 to 19 h at 37 °C.

**Table 1:** MICs for persulcatusin (IP) against *E. faecalis* and *E. faecium* strains.

Bacterial species	Strain ID	Collection year	Geographic location	Isolation source	Vancomycin resistance	Resistance gene	MIC (μg/mL)
*E. faecalis*	ATCC49533	Unknown	Wisconsin, USA	Blood	−	−	40
	VRE.fl-1	2011	Villa Clara, Cuba	Blood	+	*vanA*	20
	VRE.fl-2	1998	Kagoshima, Japan	Feces (carrier)	+	*vanA*	40
	VRE.fl-3	2012	Holguin, Cuba	Blood	+	*vanA*	40
	VRE.fl-4	2001	Havana, Cuba	Urine	+	*vanB*	40
	VRE.fl-5	1997	Tokyo, Japan	Feces (carrier)	+	*vanB*	40
	VRE.fl-6	1999	Nagano, Japan	Feces (carrier)	+	*vanB*	20
*E. faecium*	ATCC6057	Unknown	Unknown	Dairy products	−	−	1.25
	VRE.fm-1	2013	Santiago de Cuba, Cuba	Blood	+	*vanA*	0.625
	VRE.fm-2	1997	Kagoshima, Japan	Urine (urinary tract infection)	+	*vanA*	2.5
	VRE.fm-3	1998	Kagoshima, Japan	Wound pus (abscess)	+	*vanA*	0.625
	VRE.fm-4	2010	Havana, Cuba	Blood	+	*vanA*	1.25
	VRE.fm-5	2000	Akita, Japan	Feces (carrier)	+	*vanB*	2.5

### Peptides used in this study

**Figure 1:**
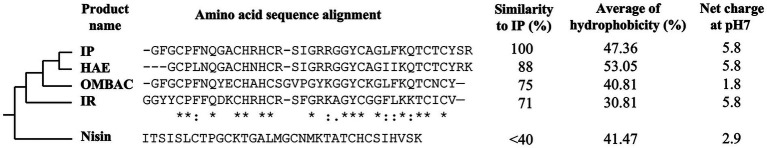
Amino acid sequences and characteristics of the peptides used in this study. The neighbor-joining (NJ) tree was constructed based on the multiple sequence alignment of persulcatusin (IP) from *Ixodes persulcatus*, HAE from *Haemaphysalis longicornis*, OMBAC from *Ornithodoros moubata*, IR from *Ixodes ricinus*, and nisin from *Lactococcus lactis*.

In the present study, we used four tick-AMPs: IP (from *Ixodes persulcatus*), HAE (from *Haemaphysalis longicornis*), OMBAC (from *Ornithodoros moubata*), and IR (from *Ixodes ricinus*). These peptides share 71 to 88% amino acid sequence similarity with IP and exhibit comparable hydrophobicity and net charge profiles at pH 7.0 (Figure 1). Nisin, a bacterial peptide derived from *Lactococcus lactis*, was included for comparison. Their amino acid sequences have been previously reported (Nakajima et al., 2002; Tsuji et al., 2007; Takagi et al., 2012). Multiple amino acid sequence alignment and construction of a neighbor-joining (NJ) tree were performed using the ClustalW program available via the GenomeNet website with default parameters^1^ (Thompson et al., 1994). Average of hydrophobicity (%) and net charge at pH7 were calculated by previously reported methods (Gomi et al., 2004; Lear and Cobb, 2016). Buforin II was used exclusively for the DNA-binding assay (Park et al., 1998).

### Peptide synthesis and purification

All peptides were synthesized using the solid-phase method, as previously detailed (Isogai et al., 2011). The peptides were purified by reverse-phase high-performance liquid chromatography (RP-HPLC) using a YMC-A 302 column (YMC Co., Ltd., Kyoto, Japan) with a Model LC-8A (Shimadzu Corporation, Kyoto, Japan). Final product purity and identity were verified by electrospray ionization mass spectrometry, and the peptides were obtained as trifluoroacetate salts. These peptides were dissolved in Hanks’ Balanced Salt Solution (HBSS; GIBCO, Grand Island, NY, United States) at pH 7.4 and stored at −20 °C until use.

### Determination of minimum inhibitory concentration of the AMPs against enterococcal strains

The antimicrobial activities of the five test agents (IP, IR, HAE, OMBAC, and nisin) and vancomycin (Sigma Chemical Co., St. Louis, MO, United States) were evaluated. MIC_90_ values were determined using the broth microdilution method, as previously reported (Miyoshi et al., 2016). Each agent was first dissolved in HBSS at a concentration of 1 mg/mL and then serially diluted in BHI broth. Fifty microliters of these solutions were added to each well of a 96-well microplate to achieve a nine-step concentration range (80, 40, 20, 10, 5, 2.5, 1.25, 0.625, and 0.313 μg/mL). Bacterial cultures were adjusted to an optical density at 660 nm (OD660) of 0.5 and subsequently diluted in BHI broth to a final concentration of 0.5–2.5 × 10^5^ CFU/mL. A 50 μL aliquot of this bacterial suspension was inoculated into each well containing 50 μL of the peptide solution, resulting in a total volume of 100 μL per well. Consequently, the final testing concentrations of the agents were 40, 20, 10, 5, 2.5, 1.25, 0.625, 0.313, and 0.156 μg/mL. For the growth control, 50 μL of the bacterial suspension was added to a mixture of 40 μL BHI broth and 10 μL HBSS. A sterility control (blank) was prepared with 100 μL of BHI broth. The plates were incubated at 37 °C for 24–36 h. The incubation period was chosen to comply with the CLSI recommendation of 24-h incubation for detecting vancomycin resistance in enterococci, and was further extended to 36 h to account for potential growth-retarded subpopulations or heteroresistant cells within the clinical isolates, thereby ensuring the accuracy of the MIC determinations (Matsuo et al., 2011; Singh et al., 2017). Bacterial growth was quantified by measuring the OD660 using a Synergy HT multi-mode microplate reader (BioTek, Winooski, VT, United States). The MIC_90_ was defined as the lowest concentration of the agent that reduced bacterial growth by >90% compared to the growth control. MIC values of *S. aureus* strain VRS1 were cited from a previous report by Miyoshi et al. (2017). Statistical significance was evaluated using the Wilcoxon rank sum test.

### Killing assay

We performed a killing assay using five representative strains: *E. faecalis* ATCC49533 (vancomycin-susceptible), *E. faecium* ATCC6057 (vancomycin-susceptible), *E. faecalis* VRE.fl-1 (vancomycin-resistant), *E. faecium* VRE.fm-1 (vancomycin-resistant), *S. aureus* JE2 (vancomycin-susceptible), and *S. aureus* VRS1 (vancomycin-resistant). The killing assays were performed at a concentration of 5 × MIC. This concentration was selected based on our previous report (Miyoshi et al., 2017). Pre-cultured bacterial cells were diluted to 1.0–5.0 × 10^5^ CFU/mL and treated with IP at a final concentration of 5 × MIC: 200 μg/mL for *E. faecalis* ATCC49553, 6.25 μg/mL for *E. faecium* ATCC6057, 100 μg/mL for *E. faecalis* VRE.fl-1, 3.125 μg/mL for *E. faecium* VRE.fm-1, 20 μg/mL for *S. aureus* JE2, and 10 μg/mL for *S. aureus* VRS1. The mixtures were incubated at 37 °C for 0, 15, 30, and 60 min. Following incubation, 100 μL aliquots were spread onto BHI agar plates (Shimadzu Diagnostics Corporation, Tokyo, Japan). The plates were incubated at 37 °C for 24 h, and viable colonies were counted. The results were expressed as a percentage relative to the control. For statistical analysis, the assay for *S. aureus* strains (*n* = 2) was performed as a positive control for IP’s rapid bactericidal activity, while the four *Enterococcus* strains were evaluated as a group (*n* = 4). Significant differences in survival rates between the *Enterococcus* spp. and *S. aureus* were determined using Welch’s t-test at each time point.

### Scanning electron microscopy observation

Enterococcal and staphylococcal cells in the mid-log phase were resuspended to a concentration of 1.0 × 10^8^ CFU/mL in Dulbecco’s phosphate-buffered saline (DPBS; GIBCO, Grand Island, NY, United States) and incubated with IP at 5 × MIC for 30 min at 37 °C. Bacterial cells treated with HBSS alone served as a negative control. The mixtures were harvested by centrifugation (1,000 × g for 10 min), and the resulting bacterial pellets were fixed in 2% glutaraldehyde (Wako Pure Chemical Industries, Osaka, Japan) for 2 h at 4 °C. The samples were subsequently dehydrated through a graded ethanol series, sputter-coated with a palladium alloy, and examined using a Hitachi SU8000 scanning electron microscopy (SEM) (Hitachi High-Tech Corporation, Tokyo, Japan).

### Localization of IP

Synthetic IP was conjugated with 5-carboxyfluorescein (FAM) by Cosmo Bio Co., Ltd. (Tokyo, Japan) using FAM (Scrum Co., Ltd., Tokyo, Japan). Bacterial cells were examined 1 and 6 h after treatment with FAM-IP (40 μg/mL). Initial staining patterns were visualized using a BZ-8100 fluorescence microscope (Keyence, Tokyo, Japan). For detailed localization analysis and tracking, bacterial suspensions (OD660 = 0.5, 30 μL) were incubated with FAM-IP (1 mg/mL, 30 μL) for 1 h at 37 °C. Following incubation, the cells were washed with HBSS, applied onto coverslips, and fixed with 2% glutaraldehyde. The samples were examined using a dark-field microscope (BX53, SPlan 40× objective, NA 0.75; Olympus, Tokyo, Japan) equipped with an epi-fluorescence system (U-FBNA narrow filter; Olympus). Images were recorded using a CCD camera (WAT-910HX; Watec Co., Yamagata, Japan), and bacterial tracking was performed using an ImageJ-based system (National Institutes of Health, Bethesda, MD, United States). To quantitatively analyze the intracellular localization of IP, we calculated the localization index for each bacterial cell. Fluorescence intensity profiles were obtained along the cross-section of the cell-body center (51 coordinates in total). Based on the observed fluorescence patterns, the cell periphery and cytosol regions were defined as coordinates 20–22 and 31–33 (cell periphery) and 25–28 (cytosol). The IP-localization index was defined as the ratio of the mean fluorescence intensity of the cell periphery to that of the cytosol, where values approaching 1 indicate uniform distribution, while higher values reflect restricted penetration and primary localization at the cell periphery. This index was calculated for 20 individual cells by bacterial species (19 for *E. faecalis* after excluding an outlier). To ensure an unbiased evaluation of the IP-localization index, 20 cells per strain were randomly selected at 400× magnification, where fluorescent signals were not discernible. Statistical significance was evaluated using the Wilcoxon rank sum test.

### DNA-binding assay

The DNA-binding characteristics of IP to pUC19 plasmid DNA were evaluated based on the method described by Park et al. (1998). Gel-retardation experiments were performed using 100 ng of pUC19 plasmid DNA (Takara Bio Inc., Shiga, Japan) combined with increasing concentrations of the peptides. A 12 μL aliquot of each mixture was applied to a 1% agarose gel for electrophoresis in Tris-borate-EDTA (TBE) buffer (Takara Bio Inc., Shiga, Japan). Buforin II, a 21-amino acid AMP derived from Buforin I of the Asian toad (*Bufo bufo gargarizans*), was used as a positive control (Park et al., 1998).

## Results

### Antimicrobial activity of IP against enterococcal strains

As shown in Table 1, IP exhibited growth-inhibitory activity against *E. faecium* strains, regardless of their vancomycin resistance profiles. IP showed low MIC values for *E. faecium* strains (ranging from 0.625 to 2.5 μg/mL), which were significantly lower than the values observed for *E. faecalis* strains (ranging from 20 to 40 μg/mL) (*p* = 0.00125). These findings suggest that the growth-inhibitory activity of IP is species-dependent rather than strain-dependent, and importantly, it is independent of vancomycin resistance profile. Based on these results, we selected four representative strains for further assays: *E. faecalis* ATCC49533 (VSEfl), *E. faecalis* VRE.fl-1 (VREfl), *E. faecium* ATCC6057 (VSEfm), and *E. faecium* VRE.fm-1 (VREfm).

Using the selected enterococcal strains, we compared the MIC values of IP with those of other cationic AMPs and vancomycin (Table 2). IP consistently demonstrated superior growth-inhibitory activity compared to the other AMPs tested. Notably, the activity of IP against both VSEfm and VREfm strains was comparable to that of vancomycin against VSE strains.

**Table 2:** MICs for various AMPs against VSE, VRE and VRSA strains.

Bacteria strain		MIC (μg/mL)					
		IP	IR	HAE	OMBAC	Nisin	Vancomycin
*E. faecalis* ATCC49533	(VSEfl)	40	>40	>40	>40	40	1.25
*E. faecium* ATCC6057	(VSEfm)	1.25	20	>40	20	10	1.25
*E. faecalis* VRE.fl-1	(VREfl)	20	40	>40	>40	10	>40
*E. faecium* VRE.fm-1	(VREfm)	0.625	10	>40	10	10	>40
*S. aureus* VRS1	(VRSA)	2	32	>32	8	16	>32

IP, persulcatusin; IR, tick antimicrobial peptide from *Ixodes ricinus*; HAE, tick antimicrobial peptide from *Haemaphysalis longicornis*; OMBAC, tick antimicrobial peptide from *Ornithodoros moubata*; Nisin, a bacterial peptide derived from *Lactococcus lactis*; VSEfl, vancomycin-susceptible *Enterococcus faecalis*; VSEfm, vancomycin-susceptible *Enterococcus faecium*; VREfl, vancomycin-resistant *Enterococcus faecalis*; VREfm, vancomycin-resistant *Enterococcus faecium*; VRSA, vancomycin-resistant *Staphylococcus aureus*.

MIC values of *S. aureus* VRS1 were cited from a previous report by Miyoshi et al. (2017).

### Time-dependent rapid antimicrobial activity of IP against enterococci and *Staphylococcus aureus*

**Figure 2:**
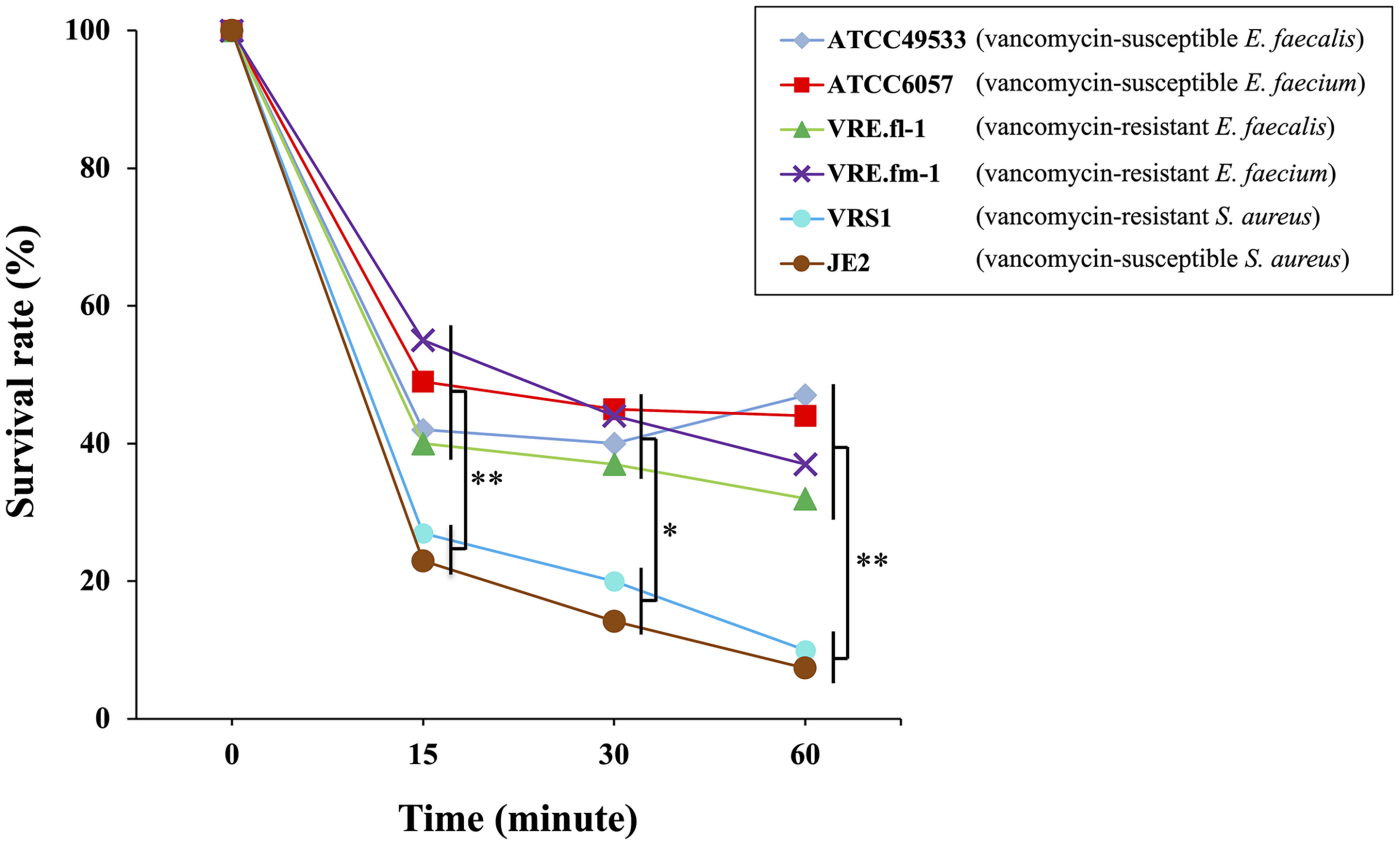
Survival rates of vancomycin-susceptible *E. faecalis* ATCC49553, vancomycin-susceptible *E. faecium* ATCC6057, vancomycin-resistant *E. faecalis* VRE.fl-1, vancomycin-resistant *E. faecium* VRE.fm-1, vancomycin-susceptible *S. aureus* JE2, and vancomycin-resistant *S. aureus* VRS1 strains treated with persulcatusin (IP). Survival rates were evaluated after 15, 30, and 60 min of exposure to 5 × MIC of IP for each strain (200, 6.25, 100, 3.125, 20, and 10 μg/mL, respectively). Survival rates of each strain are individually plotted, and statistical significance was evaluated by comparing the *Enterococcus* spp. (*n* = 4) and the *S. aureus* strains (*n* = 2); ** indicates *p* < 0.01; * indicates *p* < 0.05 (Welch’s t-test).

To evaluate the rapid bactericidal activity of IP against *Enterococcus* strains, we performed a killing assay using two *S. aureus* strains as positive controls. The assay was conducted with incubation times of 0, 15, 30, and 60 min (Figure 2). At a concentration of 5 × MIC, the viable cell counts of both *E. faecalis* and *E. faecium* decreased by 40 to 60% within 15 min regardless of their vancomycin-resistance profiles. However, the number of viable cells remained stable between 15 and 60 min (*p* > 0.05). In contrast, the viabilities of the *S. aureus* strains decreased gradually and continuously between 15 and 60 min (*p* = 0.0521), with 90% of the cells killed by the end of the experiment. Statistical analysis confirmed that the survival rates of all tested *Enterococcus* strains were significantly higher than those of *S. aureus* at 15, 30, and 60 min (*p* < 0.01, *p* < 0.05, and *p* < 0.01, respectively). Thus, the bactericidal activity of IP was potent, rapid, and time-dependent against *S. aureus*, whereas it was lower and independent of exposure time against *E. faecalis* and *E. faecium*.

### Ultrastructural alterations of enterococcal cells by IP exposure

**Figure 3:**
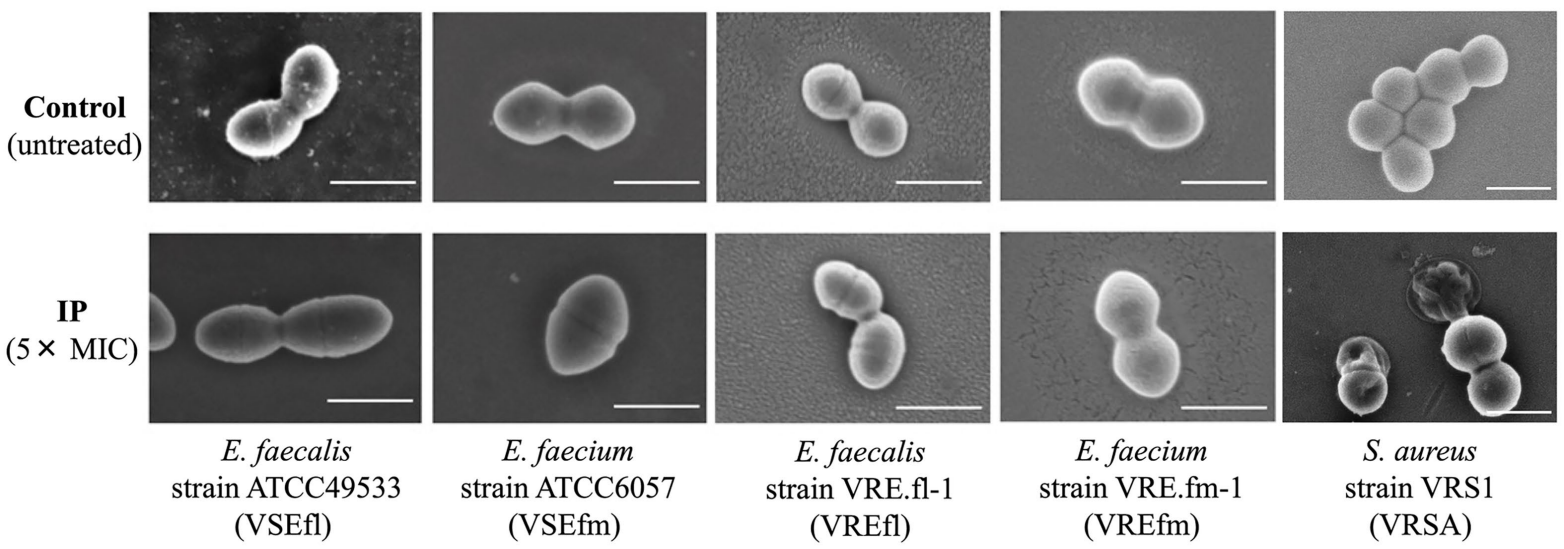
Morphological cell changes in enterococcal species and *S. aureus* induced by persulcatusin (IP) exposure. Scanning electron microscopy (SEM) images of vancomycin-susceptible *E. faecalis* ATCC49533, vancomycin-susceptible *E. faecium* ATCC6057, vancomycin-resistant *E. faecalis* VRE.fl-1, vancomycin-resistant *E. faecium* VRE.fm-1, and vancomycin-resistant *S. aureus* VRS1 strains treated with or without IP (5 × MIC) for 30 min. While the VRS1 strain showed evident structural alterations and cell crumpling, the enterococcal cells maintained smooth surfaces comparable to those of the untreated control, regardless of their species or vancomycin-resistance profile. Scale bars represent 1 μm.

We observed the morphology of enterococcal and staphylococcal strains treated with IP at 5 × MIC for 30 min (Figure 3). The *S. aureus* VRS1 strain exhibited profound structural damage, including extensive membrane disruption and leakage of intracellular contents. In contrast, the surfaces of all tested enterococcal cells remained smooth and intact, with no signs of membrane disruption, regardless of their vancomycin-resistance profile. Although IP-induced lysis in *S. aureus* VRS1 led to the rapid loss of identifiable cellular morphology, which precluded a reliable quantitative assessment of membrane-disrupted versus intact cells, the morphological contrast between the compromised *S. aureus* and the intact enterococcal cells was consistently observed across multiple fields (Supplementary Figure S1).

### Localization of IP in bacterial cells

**Figure 4:**
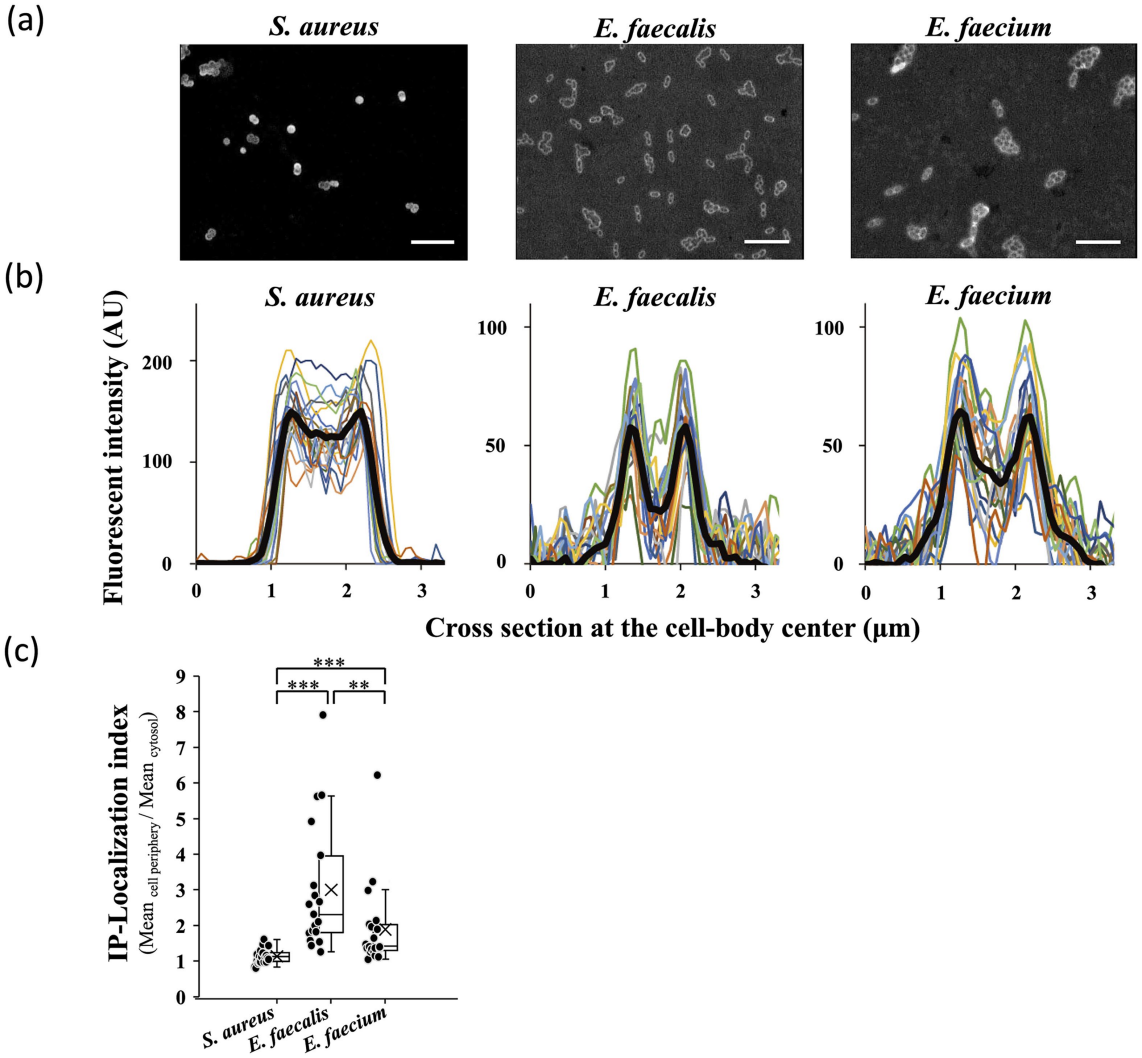
Localization of persulcatusin (IP) in vancomycin-resistant *S. aureus* VRS1, vancomycin-resistant *E. faecalis* VRE.fl-1, and vancomycin-resistant *E. faecium* VRE.fm-1 strains. **(a)** Epifluorescence images of *S. aureus*, *E. faecalis*, and *E. faecium* cells treated with FAM-labeled IP. Scale bars represent 20 μm. **(b)** Fluorescent intensity profiles were measured across the center of individual cell bodies. Thin colored lines represent the intensity profiles of individual cells, while thick black lines represent the average profiles of 20 cells. **(c)** Statistical comparison of IP localization patterns across bacterial species. The IP-localization index was calculated as the ratio of the mean fluorescence intensity at the cell periphery to that in the cytosol (*n* = 20 per species). Data are presented as box-and-whisker plots with individual data points overlaid; the cross and the horizontal line within each box indicate the mean and the median, respectively. Statistical significance was evaluated using the Wilcoxon rank-sum test for pairwise comparisons; *** indicates *p* < 0.001; ** indicates *p* < 0.01; * indicates *p* < 0.05.

To determine whether the differences between *Enterococcus* and *S. aureus* in bactericidal kinetics and membrane-disruptive activity in killing assay and SEM observations were related to distinct subcellular localization patterns of IP, we examined the localization of IP in bacterial cells. FAM-IP clearly penetrated the cell membrane and showed a uniform distribution across the entire cell, from the periphery to the cytosol, in *S. aureus* VRS1. In contrast, fluorescence in *E. faecalis* was restricted to the cell periphery. *E. faecium* exhibited an intermediate localization pattern (Figure 4a). These results were supported by fluorescence intensity profiles across cell cross-sections (Figure 4b). Consistent with the qualitative observations, *S. aureus* exhibited the lowest IP-localization index, approaching 1 (indicative of uniform distribution), whereas *E. faecalis* showed the highest, with *E. faecium* yielding intermediate values (Figure 4c). Statistical analysis confirmed significant differences among all three species for each pairwise comparison, further validating the distinct intracellular penetration profiles.

### DNA-binding assay

**Figure 5:**
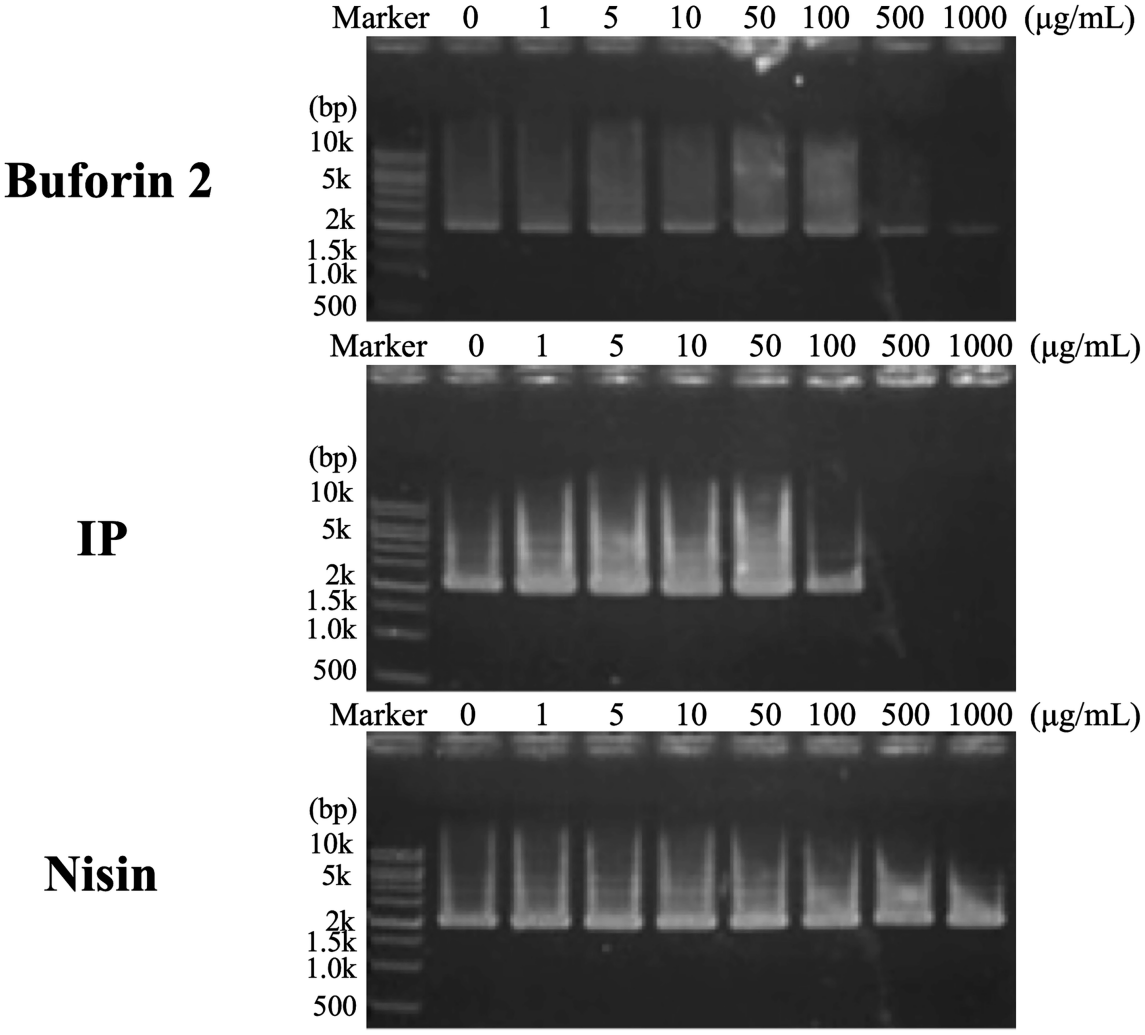
Comparison of the DNA-binding properties of persulcatusin (IP), nisin, and the positive control, buforin II. DNA-binding assays were performed to evaluate the inhibitory effects of the peptides on DNA migration using mixtures of 100 ng of pUC19 plasmid DNA and increasing concentrations of each peptide. Buforin II, a 21-amino acid antimicrobial peptide derived from *Bufo gargarizans*, was used as a positive control. IP and buforin II exhibited DNA-binding activity, whereas nisin did not. Full-length gel images are provided in Supplementary Figure S2.

The growth-inhibitory effects of IP in *S. aureus*, *E. faecium*, and *E. faecalis* correlated with the efficiency of the intracellular penetration. This correlation suggests that intracellular permeabilization and subsequent interaction with potential intracellular targets are key determinants of the differences in IP activity among these three species. To test this hypothesis, we performed a DNA-binding assay. As shown in Figure 5, IP inhibited DNA migration at concentrations of 500 μg/mL and above. This indicates that IP binds to DNA, demonstrating an activity comparable to that of the known DNA-binding peptide buforin II (Park et al., 1998), and more potent than that of nisin.

## Discussion

A key finding of the present study was that IP exhibits species-dependent antimicrobial mechanisms, shifting from the membrane-disruptive activity observed in *S. aureus* to intracellular targeting, such as DNA-binding, in *E. faecium*. In contrast, both mechanisms appeared largely absent in *E. faecalis*, resulting in its significantly reduced susceptibility to IP. While previous reports suggested that individual AMPs could possess multiple mechanisms of antibacterial action (Marr et al., 2006; Garvey, 2023; Alzain et al., 2025), our results suggest that IP shares this characteristic, modulating its primary mode of action depending on the bacterial species. The mechanistic flexibility of IP against Gram-positive nosocomial bacteria observed in this study suggests that IP could serve as a promising scaffold for developing next-generation therapeutics aimed at overcoming the problem of AMR.

We previously reported that the rigid, disulfide-stabilized framework of IP optimizes its cationic and amphipathic properties (Miyoshi et al., 2016). While this structure induces rapid membrane disruption in *S. aureus*, the present study demonstrates that such rapid bactericidal activity is absent against *Enterococcus* species. In our killing assays, even treatment with IP at 5 × MIC failed to achieve rapid sterilization, with approximately 40% of the enterococcal population remaining viable. The inability of IP to disrupt the enterococcal membrane, in contrast to the activity observed in *S. aureus* (Miyoshi et al., 2017), likely stems from the inherent resistance of *Enterococcus* species to membrane-active agents. It has been reported that this resistance is actively maintained by complex regulatory systems such as LiaFSR, which orchestrate membrane lipid remodeling, including the redistribution of cardiolipin, in response to envelope stress (Arias et al., 2011; Palmer et al., 2011). This suggests that by dynamically maintaining membrane integrity and potentially sequestering peptide molecules, the regulatory response prevents IP from achieving the critical density required for membrane lysis in *Enterococcus* species. Crucially, in *E. faecium*, IP penetrates the cells without disrupting the membrane and exhibits growth-inhibitory activity. Conversely, the densely cross-linked cell wall of *E. faecalis* may act as a trap (Signoretto et al., 2000), preventing intracellular permeabilization by IP and resulting in higher MIC values. These structural variations in the cell wall likely account for the species-specific differences in growth-inhibitory activity observed between these two enterococcal species.

Our results demonstrate that IP activity remains unaffected by *vanA*- or *vanB*-mediated vancomycin resistance mechanisms and that IP exerts growth-inhibitory effects on *E. faecium* through a mechanism independent of membrane disruption. We previously reported that the growth of the vancomycin-intermediate *S. aureus* (VISA) strain Mu50, which is characterized by significantly thickened cell walls (Hiramatsu et al., 1997; Cui et al., 2003), was inhibited by IP exposure without membrane disruption (Miyoshi et al., 2017). This phenotype is consistent with observations in *E. faecium* in the present study. These results suggest that when membrane disruption is ineffective, IP localizes intracellularly and exerts growth-inhibitory activity via intracellular targeting, such as DNA-binding, likely through a mechanism shared by both *E. faecium* and the VISA strain Mu50. Shimoda et al. (2024) reported multiple genes involved in cell wall synthesis and stress responses as potential determinants of IP susceptibility in *S. aureus*. These candidate genes included two-component regulatory system genes such as *vraSR* and *graSR*, which have been implicated in vancomycin-intermediate resistance and daptomycin resistance in the VISA strain Mu50 (Kuroda et al., 2003; Camargo et al., 2008; Cui et al., 2009; Hiramatsu et al., 2013; Hiramatsu et al., 2014). These findings suggest that the selective pressures exerted on bacterial cells by vancomycin and IP are similar in *S. aureus*, where membrane disruption is the primary mode of action. This is also supported by our previous observation that the VISA strain Mu50 exhibits insensitivity to IP-induced membrane disruption, indicating cross-resistance between vancomycin and IP (Miyoshi et al., 2017). Consequently, a comparative analysis of resistance to IP-induced membrane disruption between VISA and *Enterococcus* strains is essential to elucidate the fundamental determinants of IP susceptibility.

The present study, demonstrating that IP exhibits DNA-binding affinity comparable to that of buforin II (Park et al., 1998), provides critical insight into the intracellular mechanism of this peptide. AMPs, including indolicidin, buforin II, DM3, and microcin J25, exert their antibacterial effects and interfere with the transcription of specific or global metabolic pathways by binding to intracellular targets such as DNA, RNA, proteins, and ribosomes (Yuzenkova et al., 2002; Ghosh et al., 2014; Le et al., 2016; Le et al., 2017). Future studies will be essential to elucidate which specific or global metabolic pathways are subject to transcriptional interference by IP via such interactions.

Clinically, the application of IP should be guided by a strategic differentiation of the causative pathogen’s structural profile. Our findings suggest that Gram-positive multidrug-resistant bacteria can be categorized by their response to IP: those susceptible to rapid membrane disruption (e.g., MRSA and VRSA), where a potent bactericidal effect can be expected; and those characterized by intracellular translocation followed by binding to intracellular targets (e.g., VREfm and VISA strains). Crucially, in the latter group, while IP lacks rapid bactericidal action, it provides significant therapeutic value by maintaining growth-inhibitory (bacteriostatic) activity.

Regarding its clinical application, IP’s low MICs suggest its high potential as a standalone agent. On the other hand, synergy has been reported when IP is used in combination with ampicillin, tetracycline, and ciprofloxacin, whereas not with vancomycin or daptomycin (Shimoda et al., 2025). Some AMPs have been reported to exert synergistic effects with intracellularly targeted antibiotics such as macrolides and chloramphenicol by increasing membrane permeability (Yuzenkova et al., 2002; Cirioni et al., 2008; Choi and Lee, 2012). However, IP has not shown synergistic effects with these antibiotics, on the contrary; it has been suggested that co-administration with aminoglycoside antibiotics may lead to a decrease in antimicrobial activity due to the inhibition of uptake via membrane depolarization (Shimoda et al., 2025). Furthermore, given the potential cross-resistance with vancomycin and daptomycin mentioned above, combining IP with these drugs might risk promoting further resistance. Thus, future studies will be necessary to identify optimal combinations to ensure IP’s effective clinical implementation.

The effectiveness of IP against *E. faecalis* (including VREfl) is limited, as evidenced by the high MIC values observed in this study. Previous studies of bloodstream infections have reported that vancomycin resistance in *E. faecalis* remains relatively low at 9.8%, whereas it reaches a staggering 80% in *E. faecium* (Werner et al., 2008; World Health Organization, 2022, 2024). Consequently, the latter, where the drug’s clinical utility is severely compromised, represents a more critical clinical concern, as reflected by the WHO’s designation of VREfm as a high-priority threat (Tacconelli et al., 2018b). Given that the antimicrobial efficacy of IP against *E. faecium* is comparable to that of vancomycin against vancomycin-susceptible strains, regardless of vancomycin resistance profiles, IP represents a highly promising candidate specifically optimized to address the critical VREfm crisis.

This study has some limitations to be considered. Our evaluation was limited to *vanA* and *vanB* genotypes. Therefore, further testing against a broader range of resistance mechanisms, such as the *vanC* genotype, is required. The small sample size limits the generalizability of our findings. Further investigations involving a wider range of enterococcal strains are necessary to confirm whether IP’s growth-inhibitory activity is *E. faecium* species-specific. Additionally, MICs were determined using BHI broth instead of the CLSI-recommended Mueller–Hinton broth. BHI was used to ensure consistency with our previous studies and to support the stable expression of vancomycin-resistant phenotypes (Matsuo et al., 2011; Singh et al., 2017). However, this methodological difference should be considered when comparing our MIC values with those from standardized clinical microbiological testing.

In conclusion, the present study suggested that IP exhibits species-specific growth-inhibitory activity against *E. faecium* rather than *E. faecalis*, and that IP has dual modes of action against Gram-positive bacteria: membrane-disrupting activity, with cross-resistance to vancomycin-intermediate resistance in the VISA strain Mu50, and alternative intracellular targeting activity, possibly mediated by peptide-DNA interactions. Our findings emphasize the clinical potential of IP as a targeted strategy to combat AMR, particularly for the control of nosocomial infections caused by high-priority Gram-positive multidrug-resistant bacteria, such as VREfm, MRSA, and VISA.

## Data Availability

The original contributions presented in the study are included in the article/Supplementary material, further inquiries can be directed to the corresponding author.

## Footnotes

1
https://www.genome.jp/tools-bin/clustalw

## Supplementary material

SUPPLEMENTARY FIGURE S1 Representative SEM images of bacterial cells treated with IP. Morphology of *E. faecalis*, *E. faecium*, and *S. aureus* treated with IP at 5 × MIC for 30 min. *S. aureus* (right) exhibits extensive membrane disruption and structural collapse across multiple fields of view, as observed in Figure 3. In contrast, *E. faecalis* (left) and *E. faecium* (center) consistently display intact, smooth surfaces, indicating the absence of membrane-disruptive activity in enterococcal species under IP exposure.

SUPPLEMENTARY FIGURE S2 Full-length gel images of DNA-binding assay. Agarose gel electrophoresis showing the binding activity of buforin 2, IP, and Nisin to pUC19 plasmid DNA. The binding was assessed across a concentration range of 0 to 1,000 μg/mL. These full-length gel images serve as the raw data for the cropped gel images presented in Figure 5.
