# Supplementary figures and images for: Antimicrobial activity and mechanistic insights into the tick peptide persulcatusin against vancomycin-susceptible and vancomycin-resistant Enterococcus faecium strains
Source: Front Microbiol. 2026 May 4;17:1834116. doi: 10.3389/fmicb.2026.1834116 (PMC13180884; doi:10.3389/fmicb.2026.1834116)

Supplementary Figure S1

*E. faecalis*

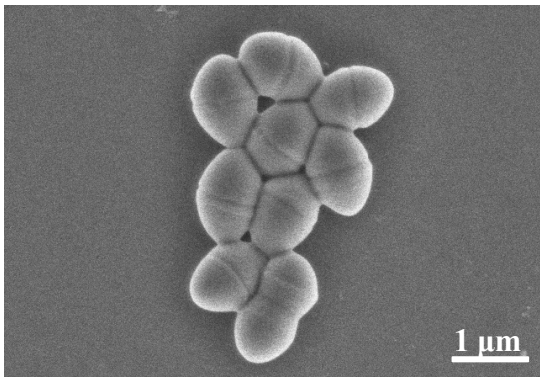

*E. faecium*

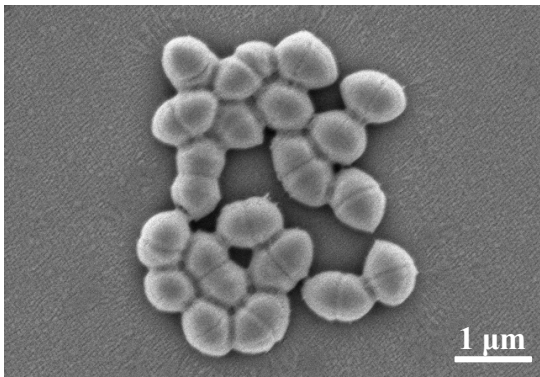

*S. aureus*

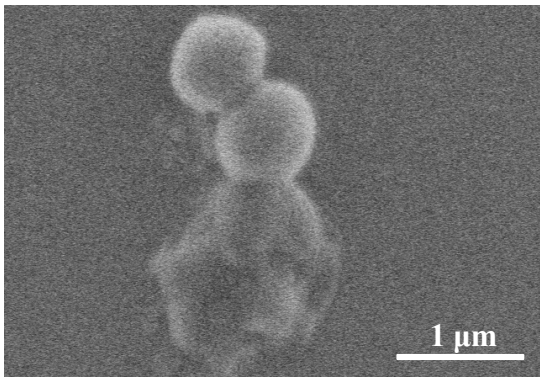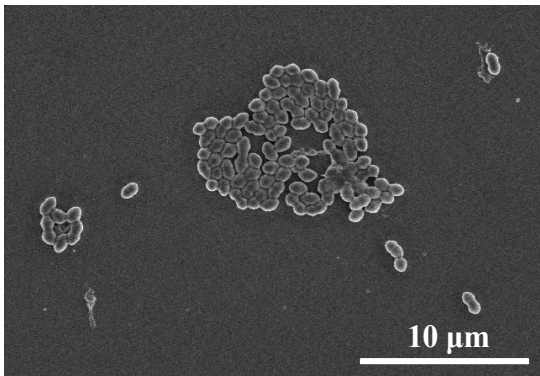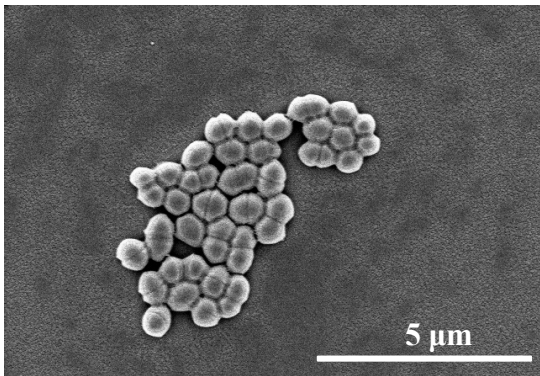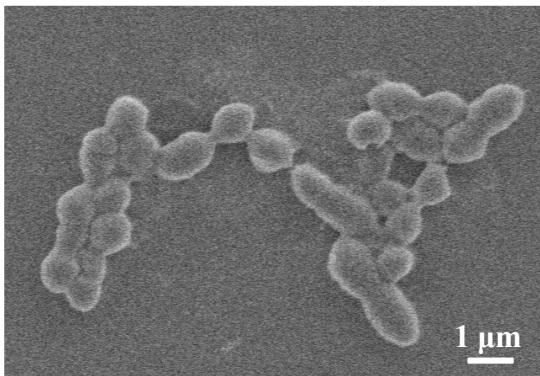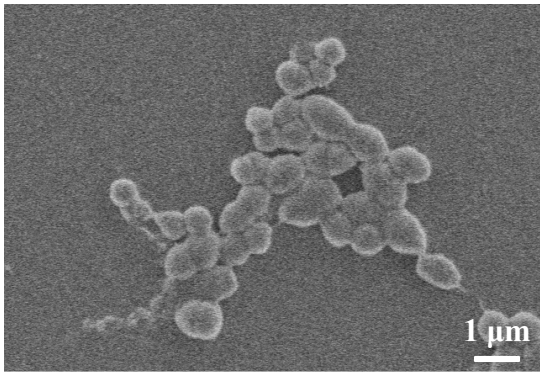

Supplementary Figure S2

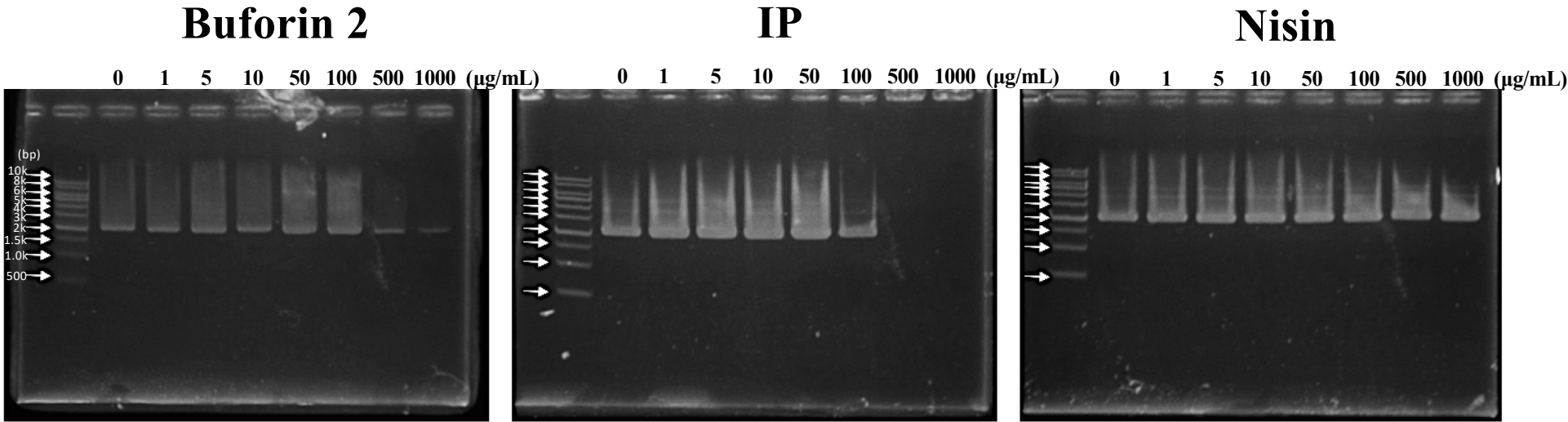

Supplement: Supplementary file 1 [file Data_Sheet_1.pdf]
